# 27. THE ROLE OF DOPAMINE IN SHAPING CIRCUITRY RELATED TO SCHIZOPHRENIA AND ADDICTION

**DOI:** 10.1093/schbul/sby014.110

**Published:** 2018-04-01

**Authors:** Anissa Abi-Dargham

**Affiliations:** Stony Brook University School of Medicine

## Abstract

**Overall Abstract:** Dopamine plays a central role in shaping circuitry within the brain, thus affecting learning and behavior. It also plays a central role in schizophrenia and addiction. This panel will examine the impact of dopaminergic signaling on specific circuits that may create special vulnerability for the emergence of comorbidity between schizophrenia and addiction. The talks will include two presentations in clinical samples using molecular and functional imaging and two presentations in animal models.

Jared Van Snellenberg will discuss connectivity of striatal substructures to the rest of the brain in drug free patients with schizophrenia and their relationship to abnormal cortical D2 signaling and psychotic symptoms. He will present recent unpublished work, motivated by pre-clinical studies with a D2 receptor over-expressing mouse model, using simultaneous multi-slice (multiband) functional MR imaging in these patients. These results suggest that unmedicated patients have altered connectivity between specific basal ganglia subnuclei, consistent with the animal model.

Nora Volkow will focus on the role of bidirectional interactions between dopamine reward system and prefrontal regions in the addicted brain with emphasis on the role of D2 receptor signaling in the striatum. She will discuss the functional impact of these interactions on reward/motivation and executive-function networks and will discuss the variables that influence D2 receptor function including genes, sleep and social stressors and how they interact with drug exposures to provide resilience or vulnerability to substance use disorders or schizophrenia. Finally, she will discuss how this knowledge can be used to tailor interventions to remediate or buffer neurocircuity dysfunction triggered by drugs and for prevention.

Bita Moghaddam will present an animal model of behaviorally induced dysfunction in the mesocortical circuit by using a task where actions are consistently rewarded but probabilistically punished. Spike activity and local field potentials are recorded during this task simultaneously from VTA and mPFC, two reciprocally connected mesocortical regions. Under no risk of punishment, a synchronous interaction at multiple time scales between PFC and VTA dopamine neurons is observed. This synchrony collapsed as a function of punishment contingency during reward-seeking actions, with risk of punishment diminishing VTA-driven neural synchrony between the two regions. These data reveal a dynamic coding scheme in VTA-mPFC neural circuits in representing aversion-based modulation of rewarded actions. These data suggest that driving VTA dopamine neurons by drugs of abuse may reverse the diminished synchrony and serve as self-medication in comorbid conditions.

Finally, Aurelio Galli will discuss the structural, functional, and behavioral insights into the dopamine dysfunction of comorbid conditions as modeled by a deletion of the SLC6A3 affecting the function of the human dopamine transporter (hDAT). Genetic variants in hDAT have been associated with neuropsychiatric disorders. An in-frame deletion in hDAT at N336 (∆N336) leads to abnormal DA homeostasis. He demonstrated these dysfunctions in brains of Drosophila melanogaster expressing hDAT ∆N336. Furthermore, these flies are hyperactive and display fear and impaired social interactions, traits associated with impaired DA neurotransmission. Insights from X-ray crystallography, electron paramagnetic resonance, molecular dynamic simulations, electrophysiology and behaviors describe how a genetic variation causes DA dysfunction resulting in combined behavioral alterations and psychostimulant use.

